# The challenges of classical galactosemia: HRQoL in pediatric and adult patients

**DOI:** 10.1186/s13023-023-02749-8

**Published:** 2023-06-02

**Authors:** Merel E. Hermans, Hedy A. van Oers, Gert J. Geurtsen, Lotte Haverman, Carla E. M. Hollak, M. Estela Rubio-Gozalbo, Annet M. Bosch

**Affiliations:** 1 Emma Children’s Hospital, Department of Pediatrics, Division of Metabolic Diseases, Amsterdam UMC Location University of Amsterdam, Meibergdreef 9, 1105 AZ Amsterdam, The Netherlands; 2 Inborn Errors of Metabolism, Amsterdam Gastroenterology Endocrinology Metabolism, Amsterdam, The Netherlands; 3 Emma Children’s Hospital, Child and Adolescent Psychiatry and Psychosocial Care, Amsterdam UMC Location University of Amsterdam, Meibergdreef 9, Amsterdam, The Netherlands; 4 Child Development, Amsterdam Reproduction and Development, Amsterdam, The Netherlands; 5 Mental Health and Quality of Care, Amsterdam Public Health, Amsterdam, The Netherlands; 6 Department of Medical Psychology, Amsterdam Neuroscience Degeneration, Amsterdam UMC Location University of Amsterdam, Meibergdreef 9, Amsterdam, The Netherlands; 7 Amsterdam Public Health, Mental Health and Digital Health, Amsterdam, The Netherlands; 8 Division of Endocrinology and Metabolism, Department of Internal Medicine, Amsterdam UMC Location University of Amsterdam, Meibergdreef 9, Amsterdam, the Netherlands; 9 Department of Pediatrics and Laboratory Genetic Metabolic Diseases, Maastricht University Medical Center, Maastricht, The Netherlands

**Keywords:** Classical galactosemia, GALT deficiency, HRQoL, PROMs, PROMIS, Psychosocial functioning

## Abstract

**Background:**

Classical galactosemia (CG), an inborn error of galactose metabolism, results in long-term complications including cognitive impairment and movement disorders, despite early diagnosis and dietary treatment. Two decades ago, lower motor-, cognitive- and social health related quality of life (HRQoL) was demonstrated in pediatric and adult patients. Since then, the diet has been relaxed, newborn screening was implemented and new international guidelines resulted in major changes in follow-up. The aim of this study was to assess HRQoL of CG by means of online self- and/or proxy-HRQoL-questionnaires focusing on the main areas of concern of CG (i.e. anxiety, depression, cognition, fatigue, social- and upper extremity function) within the patient-reported outcomes measurement information system (PROMIS®) and generic HRQoL-questionnaires (TAPQOL, TACQOL, TAAQOL).

**Results:**

Data of 61 Dutch patients (aged 1–52 years) were collected and compared to available Dutch or US reference populations. On the PROMIS-questionnaires, children reported more fatigue (*P* = 0.044), lower function in upper extremities (*P* = 0.021), more cognitive difficulties (*P* = 0.055, *d* = 0.56) and higher anxiety (*P* = 0.063, *d* = 0.52) than reference children although the latter findings remained non-significant. Parents of CG patients reported lower quality of peer relationships of their children (*P* < 0.001). Both children and parents reported lower cognitive functioning (*P* = 0.005, *P* = 0.010) on the TACQOL. Adults reported on PROMIS domains lower cognitive functioning (*P* = 0.030), higher anxiety (*P* = 0.004) and more fatigue (*P* = 0.026). Cognitive difficulties were reported on the TAAQOL by adults (*P* < 0.001), as well as physical-, sleeping and social difficulties.

**Conclusions:**

CG remains to impact the HRQoL of pediatric and adult patients negatively on several domains including cognition, anxiety, motor function and fatigue. A lower social health was mainly reported by parents, and not by patients themselves. The Covid-19 pandemic might have amplified the results on anxiety although higher levels of anxiety fit pre-pandemic findings. The reported fatigue is a new finding in CG. Because the effect of lockdown fatigue could not be eliminated and fatigue is a frequent finding in patients with chronic disorders, future studies are warranted. Clinicians and researchers should be attentive to both pediatric and adult patients, and the age-dependent difficulties they might encounter.

**Supplementary Information:**

The online version contains supplementary material available at 10.1186/s13023-023-02749-8.

## Background

Classical galactosemia (CG; OMIM 230400) is an autosomal recessive inborn error of galactose metabolism with an incidence of 1:53.000 in the Netherlands [1]. A severe deficiency of the enzyme galactose-1-phosphate-uridyltransferase (*GALT*; EC 2.7.7.12) results in a life-threatening disease in newborns after the ingestion of galactose from breastmilk or infant feeding. Currently the only available treatment is a galactose-restricted diet which resolves the critical symptoms in affected newborns, but does not prevent long-term complications. Even with immediate or early treatment due to family screening or newborn screening and independent of illness in the newborn period, many patients develop long-term complications. These include a developmental delay in motor function, speech- and language difficulties, a cognitive impairment, movement disorders, and primary ovarian insufficiency in female patients [2]. The underlying mechanism of these long-term complications is still poorly understood. The persistent elevation of metabolites including galactose-1-phosphate (Gal-1P) due to endogenous production of galactose has been demonstrated to affect galactosylation [3, 4]. However, in our cohort Gal-1P and *N*-glycan variations could not predict clinical outcome [2]. The severity of the outcome differs tremendously between patients, with total IQ’s ranging from 45 to 103 and neurological outcome ranging from severe dystonia to fully normal [2, 5, 6]. Social participation and adjustment to society are lower, indicated by higher levels of unemployment [7] and fewer milestones in the psychosexual and social domain [8, 9]. Living with a chronic disorder and with this prognostic uncertainty regarding outcome severity is a burden for many parents and children with CG [10]. Previous studies demonstrated a lower Health Related Quality of Life (HRQoL) in both children and adults with CG [8, 10, 11]. Children with CG reported a lower HRQoL in the domain of cognitive function, while parents of children with CG reported a lower HRQoL in the domain of both motor- and cognitive function of their children [10]. In adult patients a lower HRQoL in the domains of cognitive function, social function and emotional wellbeing was found in comparison to the general population [10] and compared to patients with another metabolic disorder with dietary restrictions (Phenylketonuria; PKU) [8, 11]. The lower HRQoL in comparison to PKU-patients shows that HRQoL is not fully explained by the presence of a chronic disease and a lifelong diet. It is more likely that in CG the long-term complications influence the HRQoL of patients. In parents of CG-patients, a normal HRQoL was found in a previous study [12].

Since our previous HRQoL study in 2004 [10], and especially with the implementation of the international guidelines, major changes in treatment and follow up have been implemented. The diet has been relaxed, allowing patients an unrestricted intake of fruit and vegetables and food products with minor traces of galactose [13]. Social difficulties were discussed with patients and parents and social interaction of patients has been stimulated. Neurological and cognitive outcomes of patients are regularly evaluated with standardized screening allowing an early intervention if necessary.

In the Netherlands, the start of the newborn screening (NBS) in 2007 resulted in an expansion of the spectrum of galactosemia patients since new patients were detected who did not demonstrate CG-related illness [1]. These “variant” patients show previously unknown genotypes and have some residual galactose oxidation. The phenotype seems milder than in the classical patients and it is yet unclear whether these patients are at risk for the same long-term complications as the “classic” patients with severe enzyme deficiency. HRQoL of this group has not yet been investigated.

Up till now, HRQoL in CG has been solely investigated by means of generic questionnaires assessing multiple domains of health. Domains are assessed with few items which all contribute equally to the domain score, limiting the reliability and validity [14]. In recent years, the Patient-Reported Outcomes Measurement Information System (PROMIS®) has been developed to address these limitations by developing item banks based on item response theory modeling taking into account the differences in item content [15].

The main aim of the current study is to assess HRQoL of the complete spectrum of CG patients, firstly by unidimensional PROMIS domains of physical, mental and social health specific to areas of concern in patients with CG, and secondly by the generic, multidimensional HRQoL questionnaires which were also administered almost 2 decades ago to describe the effects of the changes in treatment and follow up since 2004. The secondary aims are:

A. Examine the differences in HRQoL between patients diagnosed before the introduction of NBS after the presentation of clinical symptoms and patients diagnosed after family screening and/or NBS.
B. Examine the effect of outcome severity on HRQoL by evaluating the association between HRQoL and total intelligence quotient (IQ) and the difference in HRQoL in patients with good intellectual outcome (IQ ≥ 85) and poor intellectual outcome (IQ < 85).

## Results

A total of 24 children and/or their parents, 31 adult patients and 6 representatives of cognitively impaired adult patients participated, providing data on a total of 61 patients which led to an overall response rate of 51%. Assessment of the group of non-participating patients of the outpatient clinic of the Amsterdam UMC indicated no response bias towards patients with a relatively good or poor outcome. The completion rate was 97–100% for each questionnaire. Because of the presence of a second diagnosis affecting mobility, one child was excluded for the PROMIS domains of fatigue and upper extremity function, and the TAAQOL-scales gross motor functioning and pain.

The demographics of the patients are listed in Table 1. Twenty-four children were included in the study with a mean age of 8.9 years (*SD* = 5.2, *range* 1–17). Thirty-one self-reporting adult patients participated in the study with a mean age of 31.2 years (*SD* = 9.2, *range* 18–52). Additionally, six adult patients participated by means of proxy-report by their representatives with a mean age of 27.7 years (*SD* = 6.0, *range* 18–34). Eleven children had received formal intelligence assessment with a mean IQ of 82.7 (*SD* = 10.8, *range* 58–95). Formal intelligence assessment was available for 16 self-reporting adults, with a mean IQ of 76.5 (*SD* = 10.5, *range* 53–93).

**Table 1:** Demographics of all patients separated into three age categories

	*N*	All CG patients (*N* = 61)	*N*	Children age < 8 years (*N* = 9)	*N*	Children 8–17 years (*N* = 15)	*N*	Adults^#^ self-reporting (*N* = 31)	*N*	Adults with representative^##^ (*N* = 6)
Gender, %	61		9		15		31		6	
Female	32	52%	1	11%	7	47%	21	68%	3	50%
Male	29	48%	8	89%	8	53%	10	32%	3	50%
Age in years, mean (range)	61	22.1 (1–52)	9	3.2 (1–7)	15	12.4 (8–17)	31	31.2 (18–52)	6	27.7 (18–34)
GALT erythrocyte activity (%), %	52		8		15		23		6	
< 3.3%	49	94%	6	75%	14	93%	23	100%	6	100%
≥ 3.3%	3	6%	2	25%	1	13%	0	0%	0	0%
Diagnosis based on, %	61		9		15		31		6	
Clinical symptoms (pre-NBS)	33	54%	0	0%	3	20%	26	84%	4	67%
NBS	15	25%	7	78%	8	53%	0	0%	0	0%
FS	13	21%	2	22%	4	27%	5	16%	2	33%
Movement disorders, *n* (%)	20		0		7		10		3	
Present	12	60%	–		2	29%	7	70%	3	100%
Absent	8	40%	–		5	71%	3	30%	0	0%
Total IQ, mean (range)	32	75.7 (45–95)	1	58.0 (–)	10	85.2 (69–95)	16	76.5 (53–93)	5	57.8 (45–74)
TIQ < 85	23	72%	1	100%	4	40%	13	81%	5	100%
TIQ ≥ 85	9	28%	0	0%	6	60%	3	19%	0	0%

*CG* classical galactosemia, *y* years, *N* sample size, *NBS* newborn screening, *FS* family screening, *TIQ* total intelligence quotient

^**#**^Self-report

^##^Proxy-report of a limited number of questionnaires

As presented in Table 2, a large part of both the pediatric and adult patients receives or received special education in comparison to the general population [16–18]. Educational attainment is lower [19]. More adult patients live with their parents than in the general population and some live in an assisted living facility [20].

**Table 2:** Education and living situation

	CG-patients	Dutch population
Educational level, %		
Elementary school (≥ 4 years)	*N* = 55	Dutch population 2021/2022*, %
Normal education	58%	95%
Special education	42%	5%
Secondary school (≥ 12 years)	*N* = 48	Dutch population 2021/2022**, %
No secondary school	2%	Unknown
Normal education	81%	96%
Without support	62%	94%
With support	38%	6%
Special education	17%	4%
Educational attainment (≥ 15 years)^$^, %	*N* = 40	Total Dutch population (15–55 years), %
Low educational level	50%	21%
Secondary educational level	45%	38%
High educational level	5%	40%
Living situation (≥ 18 years), %	*N* = 37	Total Dutch population (18–52 years), %
Independent	57%	81%
Living with parents/caregivers	35%	18%
Assisted living	8%	1%

*CG* classical galactosemia, *N* sample size

*Current number of Dutch students in elementary school for schoolyear 2021/2022

**Current number of Dutch students in secondary school for schoolyear 2021/2022

^$^Highest completed level of education

### Pediatric patients

#### PROMIS

Fifteen children with galactosemia aged 8–18 years completed at least one of the PROMIS self-report questionnaires (Table 3). In comparison to the general pediatric population, children with galactosemia reported significant more fatigue (*P* = 0.044, *d* = 0.62) and significant lower upper extremity function (*P* = 0.021, *d* = 0.73). Children with galactosemia reported lower cognitive functioning [medium effect size, not significant (*P* = 0.055, *d* = 0.56)] and higher levels of anxiety [medium effect size, not significant (*P* = 0.063, *d* = 0.52)] than the reference children. There were no differences in depressive symptoms and the quality of peer relations.

**Table 3:** Self-, and proxy-reported health of children with galactosemia on PROMIS domains

	Children (age 8–18)					Parents or caregivers (age children 5–18)				
	Galactosemia		Reference population	*P*	*d*	Galactosemia		Reference population	*P*	*d*
	N	Mean (SD)	Mean (SD)			N	Mean (SD)	Mean (SD)		
Anxiety^^^	15	48.2 (8.1)	44.0 (10.5)^a^	0.063	0.52	16	52.3 (7.9)	50.0 (10.0)^d^	0.272	0.28
Depression^^^	14	47.9 (9.6)	45.0 (11.2)^a^	0.287	0.30	16	46.8 (6.9)	50.0 (10.0)^d^	0.080	0.47
Fatigue^^^	13	45.6 (9.3)	39.8 (12.4)^b^	0.044*	0.62	15	44.2 (6.9)	50.0 (10.0)^d^	0.006**	0.84
Cognition^+^	14	46.8 (5.7)	50.0 (10.0)^c^	0.055	0.56	16	46.4 (6.9)	50.0 (10.0)^c^	0.054	0.52
Upper extremity function^+^	13	45.3 (6.4)	50.0 (10.0)^d^	0.021*	0.73	15	45.6 (10.0)	50.0 (10.0)^d^	0.315^#^	0.31^$^
Peer relations^+^	14	47.7 (6.4)	46.9 (9.5)^e^	0.648	0.12	16	43.8 (5.0)	50.0 (10.0)^d^	< 0.001***	1.25

*N* sample size, *SD* standard deviation, *P* = *P* value, *d* = Cohen’s D; ≥ 0.20 = small effect, ≥ 0.50 = medium effect, ≥ 0.80 = large effect

*P < 0.05, **P < 0.01, ***P < 0.001

^^^Higher scores indicate more symptoms

^+^Higher scores indicate better functioning

^#^Non-parametric test

^$^Effect size calculated with median and median absolute deviation

^a^Normative sample of 2893 Dutch Children [21]

^b^Normative sample of 527 Dutch children [22]

^c^PROMIS reference sample of the US general population

^d^PROMIS reference sample of a subset of the US general population and a clinical sample

^e^Normative sample of 527 Dutch Children [23]

Sixteen parents of patients with CG aged 6–18 years, completed the PROMIS proxy-report questionnaires about their child (Table 3). In contrast to the self-report of the children, they reported significant lower levels of fatigue (*P* = 0.006, *d* = 0.84) and a significant lower quality of peer relations in comparison to parents of reference children (*P* < 0.001, *d* = 1.25). A medium effect size was found for lower cognitive functioning (*d* = 0.52), although not significant (*P* = 0.054). There were no differences reported in anxiety, depressive symptoms and upper extremity function.

To differentiate between children with the classical and the variant phenotype, the only variant patient above the age of 6 was in an exploratory manner removed from the analyses of the PROMIS-questionnaires. This did not result in a different pattern of significant results (results not shown).

#### TNO-AZL: TAPQOL and TACQOL

Mean scores and effect sizes are presented in Table 4. Parents of young patients with galactosemia (*N* = 7) reported significantly more stomach complaints (cramps and pain) in their child than parents of children of the general population (*P* < 0.001) on the TAPQOL. In the group of classical phenotype children, the level of communication was significantly lower than in the general population (*P* = 0.033).When including the only variant patient in this age cohort above the age of 1.5 years the difference was no longer significant. The other results were not altered after including the variant patients.

**Table 4:** Proxy-reported HRQoL (TAPQoL) for children with galactosemia

	Galactosemia children		General population		*P*	*r*
	N	Mean (SD)	N	Mean (SD)		
Sleep	7	75.0 (20.1)	340	82.3 (17.3)	0.313	− 0.05
Appetite	7	88.1 (10.6)	340	84.6 (13.2)	0.510	− 0.04
Lungs	7	90.5 (25.2)	340	93.6 (16.2)	0.884	− 0.01
Stomach	7	75.0 (18.6)	340	91.9 (13.8)	< 0.001***	− 0.18
Skin	7	90.5 (7.5)	340	91.8 (10.8)	0.437	− 0.04
Motor function	5	100 (0)	289	98.5 (4.4)	0.382	− 0.05
Social function	5	86.7 (21.7)	292	91.3 (15.4)	0.506	− 0.04
Problem behavior	7	73.5 (12.2)	340	67.7 (15.3)	0.291	− 0.06
Communication	5	81.3 (18.2)	285	91.7 (9.9)	0.143	− 0.09
Anxiety	7	85.7 (20.2)	340	78.3 (18.0)	0.234	− 0.06
Positive mood	7	97.6 (6.3)	340	98.7 (6.5)	0.222	− 0.07
Liveliness	7	100 (0)	340	98.0 (8.0)	0.459	− 0.04

*N* sample size, *SD* Standard deviation, *P* = *P* value of non-parametric test, *r* = Pearson *r* correlation; ≥ 0.10 = small effect, ≥ 0.30 = medium effect, ≥ 0.50 = large effect. Higher scores indicate a better quality of life

*P < 0.05, **P < 0.01, ***P < 0.001

The generic HRQoL of patients aged from 6 to 16, measured by the TACQOL questionnaire, was completed by parents (*N* = 14) and patients aged 8–16 (*N* = 12). Both the children and the parents reported lower scores on the domain of cognitive functioning than the general population (respectively *P* = 0.005 and *P* = 0.010, Table 5). Specifically, almost all children and parents reported difficulties with mathematics as indicated by the evaluation of the individual items. Moreover, children reported difficulties with understanding others, and writing. Parents reported attentional problems and difficulties with understanding schoolwork. Eliminating the variant patient did not alter the results (results not shown).

**Table 5:** Self- and proxy-reported HRQoL (TACQoL) of children with galactosemia

	Children		*P*	*r*	Parents		*P*	*r*
	Galactosemia children (*N* = 12) Mean (SD)	General population (*N* = 200) Mean (SD)			Galactosemia children (*N* = 14) Mean (SD)	General population (*N* = 200) Mean (SD)		
Physical symptoms	23.8 (5.0)	24.5 (5.7)	0.665	− 0.03	28.1 (3.7)	27.2 (4.0)	0.186	− 0.09
Motor function	29.8 (2.4)	29.8 (2.9)	0.608	− 0.04	30.0 (3.6)	30.5 (2.5)	0.771	− 0.02
Autonomy	31.1 (1.8)	31.5 (1.3)	0.651	− 0.03	30.6 (2.4)	31.5 (1.5)	0.600	− 0.04
Cognitive function	23.3 (5.8)	28.5 (3.9)	0.005**	− 0.19	24.2 (5.4)	29.0 (3.6)	0.010*	− 0.18
Social function	28.3 (4.7)	29.3 (3.2)	0.969	− 0.00	29.1 (4.7)	29.6 (2.5)	0.223	− 0.08
Positive emotions	13.1 (2.8)	13.4 (2.8)	1.000	0.00	13.8 (3.4)	14.2 (2.6)	0.342	− 0.06
Negative emotions	11.8 (2.8)	11.8 (2.6)	1.000	0.00	12.1 (3.0)	11.7 (2.5)	0.313	− 0.07

N = Sample size. SD = Standard deviation. *P* = *P* value of non-parametric test, *r* = Pearson *r* correlation; ≥ 0.10 = small effect, ≥ 0.30 = medium effect, ≥ 0.50 = large effect. Higher scores indicate a better quality of life

*P < 0.05, **P < 0.01, ***P < 0.001

### Adult patients

#### PROMIS

Adults with CG (*N* = 31) able to complete the questionnaires themselves demonstrated significant lower functioning in comparison to the total adult general population on three PROMIS domains (Table 6). The patients reported significantly higher levels of anxiety (*P* = 0.004, *d* = 0.57), lower levels of cognitive functioning (*P* = 0.030, *d* = 0.42) and higher levels of fatigue (*P* = 0.026, *d* = 0.43). The individual items of the short forms demonstrated that adult patients reported mainly slow thinking, difficulties with adding and subtracting numbers, and shifting between cognitive tasks. Remarkably, the adult patients reported higher ability to participate in social roles compared to the adult general population, indicating higher social health (*P* = 0.025, *d* = 0.94).

**Table 6:** Self-reported health of adults with galactosemia on PROMIS domains

	Galactosemia		Reference population	*P*	*d*
	N	Mean (SD)	Mean (SD)		
Anxiety^^^	30	54.8 (8.6)	49.9 (10.1)^a^	0.004*	0.57
Depression^^^	30	51.3 (7.9)	49.6 (10.0)^a^	0.255	0.21
Fatigue^^^	30	53.8 (10.8)	49.1 (10.8)^c^	0.026*	0.43
Cognition^+^	30	45.9 (9.9)	50.0 (10.0)^b^	0.030*	0.42
Physical functioning^+^	30	51.5 (7.8)	49.8 (10.8)^d^	0.247	0.22
Participation social roles^+^	31	53.2 (7.3)	50.6 (9.8)^e^	0.025^#^*	0.94^$^
Satisfaction social roles^+^	30	47.9 (7.9)	47.5 (8.3)^e^	0.761	0.06
Companionship^+^	30	50.2 (8.8)	50.0 (10.0)^b^	0.923	0.02
Emotional support^+^	30	53.0 (8.6)	50.0 (10.0)^b^	0.068	0.35
Social Isolation^^^	30	48.3 (9.9)	50.0 (10.0)^b^	0.359	0.17

*N* sample size, *SD* standard deviation, *P* = *P* value. *D* Cohen’s D; ≥ 0.20 = small effect, ≥ 0.50 = medium effect, ≥ 0.80 = large effect

*P < 0.05, **P < 0.01, ***P < 0.001

^^^Higher scores indicate more symptoms

^+^Higher scores indicate better functioning

^#^Non-parametric test

^$^Effect size calculated with median and median absolute deviation

^a^Normative sample of 1002 Dutch adults [24]

^b^PROMIS reference sample of the US general population

^c^Normative sample of 1006 Dutch adults [25]

^d^Normative sample of 1310 Dutch adults [26]

^e^Normative sample of 1002 Dutch adults [26]

Since the scores on seven PROMIS domains (i.e. anxiety, depression, cognition, fatigue, physical functioning, participation with social roles and social isolation) were significantly related to age and the sample of the general population was older on average, the results were compared with the available reference values for different age groups (i.e. anxiety, depression, fatigue, physical functioning, participation- and satisfaction with social roles; see Additional file 1: Table S1). The patients able to complete the questionnaires themselves between the age of 18 and 34 (*N* = 22), reported no differences in quality of life, and even reported a significant higher ability to participate in social roles than the general population between 18 and 34 years old (*P* = 0.002, *d* = 0.76). The patients (*N* = 5) between the age of 35 and 44 reported significantly higher levels of anxiety (*P* = 0.048, *d* = 1.26) and lower physical functioning (*P* = 0.008, *d* = 2.21) than adults of the general population of the same age. Moreover, medium to large effect sizes were found for depressive symptoms (*d* = 0.79), fatigue (*d* = 0.89) and the satisfaction with social roles (*d* = 0.81), although the differences remained non-significant. They reported more depressive symptoms (*P* = 0.104), a higher level of fatigue (*P* = 0.118), but also a higher level of satisfaction with their social roles (*P* = 0.145). The four patients (*N* = 4) between the age of 45 and 54 years did not differ significantly from adults of the general population between 45 and 54 years old, even though medium to large effects sizes (*d* = 0.51–> 2.0) were found representing a lower functioning on all domains including satisfaction with their social roles. A large difference in cognition was apparent between the patients between 18 and 34 years old (*mean T-score* = 50.0) and the two older age-groups (*mean T-score* = 34.7 and 34.5), but could not be evaluated against age-appropriate references.

##### Proxy-report of the representatives of patients with severe cognitive impairment

The proxy report of the representatives of six patients with severe cognitive impairment unable to complete the questionnaires (mean age: 28 years, 3 females and 3 males; IQ available for 5 patients [*mean* = 58, *SD* = 10.8, *range* 45–74)] was evaluated descriptively since the measurements used were not developed for proxy report. As expected, all representatives evaluated their child’s cognitive quality of life as below average. Especially, adding and subtracting numbers was reported as difficult. Also, slowed thinking and difficulties with shifting between tasks were reported. Moreover, the two PROMIS domains on social health (i.e. ability to participate in social roles and companionship) indicated that half of the patients were struggling whilst the other half of the patients had an average score. Physical functioning was in the average range, except for one patient.

#### TNO-AZL: TAAQOL

Adult patients (≥ 16 years; *N* = 32) reported lower motor function for both gross- and fine motor function (*P* < 0.001), lower quality of sleep (*P* = 0.001), lower cognitive functioning (*P* < 0.001), lower social functioning (*P* = 0.011), lower satisfaction with their sexual activities (*P* = 0.041) and lower levels of vitality (*P* = 0.001) compared to the reference population of adults within the same age range (16–52). For cognitive functioning, memory problems were the most prevalent. The lowered quality of sleep was mainly due to problems with falling asleep. The lower levels of vitality were mainly due to higher levels of fatigue. Mean scores and effect sizes are presented in Table 7.

**Table 7:** Self-reported HRQoL (TAAQoL) of adults with galactosemia

	Galactosemia adults		General population^$^ (*N* = 2794)	*P*	*r*
	N	Mean (SD)	Mean (SD)		
Gross motor	31	79.2 (27.0)	91.1 (18.2)	< 0.001***	− 0.09
Fine motor	32	90.4 (19.8)	97.5 (10.2)	< 0.001***	− 0.09
Pain	31	73.8 (26.5)	76.5 (22.4)	0.729	− 0.01
Sleeping	32	55.5 (35.8)	76.0 (25.1)	0.001**	− 0.06
Cognitive function	32	62.9 (30.2)	86.0 (21.0)	< 0.001***	− 0.09
Social function	32	77.0 (23.9)	86.3 (18.0)	0.011*	− 0.05
Daily activities	32	75.6 (32.2)	84.4 (23.6)	0.118	− 0.03
Sexuality	32	78.5 (28.8)	87.2 (23.5)	0.041*	− 0.04
Vitality	32	50.3 (28.7)	65.9 (22.9)	0.001**	− 0.06
Happiness	32	60.4 (29.0)	66.9 (21.3)	0.553	− 0.01
Depressive moods	32	67.7 (28.0)	79.1 (19.7)	0.061	− 0.04
Agressiveness	32	90.6 (12.3)	87.4 (16.6)	0.073	− 0.03

*N* sample size, *SD* standard deviation, *P* = *P* value of non-parametric test, *r* = Pearson *r* correlation; ≥ 0.10 = small effect, ≥ 0.30 = medium effect, ≥ 0.50 = large effect. Higher scores indicate a better quality of life

*P < 0.05, **P < 0.01, ***P < 0.001

^$^General population within the same age range (16–52 years)

### Unidimensional PROMIS domains in subgroups of patients based on diagnosis

#### Subgroups of pediatric patients

Children with galactosemia diagnosed after newborn- or family screening (*N* = 12) and children diagnosed after clinical presentation (*N* = 3) did not differ significantly on PROMIS domains (see Additional file 2: Table S2). The PROMIS proxy-report of parents also did not differ significantly between patients diagnosed after family- or newborn screening (*N* = 13) and patients diagnosed after clinical presentation (*N* = 3).

#### Subgroups of adult patients

There were no significant differences between adults diagnosed after family screening (*N* = 5) and adults diagnosed after clinical presentation (*N* = 26) on PROMIS domains (see Additional file 2: Table S3).

### Association with intelligence quotient

Intelligence quotient was not associated with age (*P* = 0.187). Correlation analyses did not reveal any significant correlations between IQ and the child- and proxy report on any of the PROMIS domains (results not shown). Children with the classic phenotype with a good intellectual outcome (i.e. IQ ≥ 85; *N* = 5) did not differ significantly on the PROMIS domains from children with a poor intellectual outcome (i.e. IQ < 85; *N* = 4; see Additional file 2: Table S4). Parent proxy report on the PROMIS domains did not differ between children with good (*N* = 5) or poor intellectual outcome (*N* = 5).

In adults, significant correlations were found between the total IQ and four domains on PROMIS domains: a higher IQ corresponded to higher levels of participation in, and satisfaction with social roles, lower levels of social isolation and higher physical functioning (results not shown). A subgroup of patients with poor intellectual outcome (i.e. IQ < 85; *N* = 14) reported significantly higher levels of anxiety (*P* = 0.014), depressive symptoms (*P* = 0.003), fatigue (*P* = 0.001) and social isolation (*P* = 0.010), and lower levels of cognitive functioning (*P* = 0.001), physical functioning (*P* = 0.044), and participation in social roles (*P* = 0.006) than patients with a good intellectual outcome (i.e. IQ ≥ 85; *N* = 4; see Additional file 2: Table S5). Satisfaction with social roles, companionship and emotional support did not differ. On the TAAQOL, adult patients with poor intellectual outcome (i.e. IQ < 85; *N* = 14) reported a significantly lower level of cognitive functioning (*P* = 0.045) and less satisfaction with their sexual activity (*P* = 0.016) than patients with a good intellectual outcome (i.e. IQ ≥ 85; *N* = 5; see Additional file 2: Table S6).

## Discussion

The present study indicates that having CG negatively impacts the HRQoL of both children and adults as measured by unidimensional PROMIS domains of health which address the main areas of concern in CG and by multidimensional generic HRQoL questionnaires. Patients and/or parents specifically reported lower functioning on the domains of cognition, anxiety, motor function, fatigue and social functioning with age differences between CG-patients. Our findings are relevant for the documentation of the natural history of galactosemia. A significant improvement in self- and proxy reported health as measured by repeated evaluation with PROMIS measures could be an important parameter of effectiveness of future novel treatments.

First, parents, pediatric- and adult patients all reported lower cognitive functioning in comparison with the general population. The self- and proxy report of the pediatric PROMIS domains remained non-significant, but medium effect sizes were found as well as a significant lower HRQoL on the cognition-scale of the TACQOL. In adults both the PROMIS domain of cognition and the cognition-scale of the TAAQOL revealed lower cognitive functioning. This finding is not surprising with regard to the well-known long-term effect of CG on intellectual development [27] and the neuropsychological difficulties that CG-patients face [28, 29]. There was no specific pattern of cognitive complaints when looking at the individual items of the questionnaires, except that the majority of children, their parents and adult patients reported difficulties with mathematics. Despite the lack of age-referenced normative values for the adult PROMIS questionnaire regarding cognition, a very large difference was apparent in cognitive function between “older” (≥ 35 years) and “younger” self-reporting adult patients (< 35 years) who were able to complete the questionnaires themselves, in which the older patients reported lower cognitive functioning. This could not be objectified by lower IQ-scores in this sample of self-reporting adults. However, it is important to note that 6 of the 28 patients aged 18–34 were unable to fill-out the these questionnaires themselves due to severe cognitive impairment thus indicating cognitive difficulties in this age cohort which might have created a positive bias in the self-reporting group.

Second, all patients reported more anxiety than reference groups. Children did report more anxiety with a medium effect size, but the effect remained non-significant. Parents of these patients did not report more anxiety in comparison to parents of reference children. Children and parents did not report significant differences on the negative emotions-scale of the TACQOL as well, although not surprising with regard to the multidimensionality of this scale. In contrast, self-reporting adult patients were more anxious than the general population with differences in age groups after evaluation against age-referenced normative values as measured with PROMIS. Since anxiety could not be investigated by proxy-report in the patients with severe cognitive impairment and participating with a representative, it is unclear whether the anxiety levels in the age-group of 18–35 might have been abnormal if the severely cognitive impaired-group would have been included. The elevated levels of anxiety reported in our cohort of patients with CG may partly be due to the Covid-19 pandemic. Indeed, anxiety levels in the general population were elevated in the second year of the pandemic when our study was conducted [30, 31]. Using the same PROMIS domain of anxiety, children with galactosemia were not more anxious than children of the general population in the two time-periods surrounding the time-period of data collection in the current study [31]. However, the current findings are in line with the previous pre-pandemic findings demonstrating internalizing problems in children with CG and reports of anxiety symptoms or a diagnosed anxiety disorder in some of the adults [6, 29]. As stated in the clinical guideline [13], it is important to screen both children and adults for mental health issues.

Third, children reported lower upper extremity function on the unidimensional PROMIS domain. This result was not supported by significant differences on the motor scale of the TACQOL, although that scale might not have been specific enough for upper extremity function. Parents did not report a significant difference in upper extremity function when compared to the same reference sample. Adult patients also did not report significant differences in physical functioning on the PROMIS domain. In contrast, the generic TAAQOL did indicate physical problems, both for fine- and gross motor functioning. These results are in line with previous findings of movement disorders (mainly tremors) in pediatric and adult patients [2, 6].

Fourth, both pediatric and adult patients reported significantly more fatigue. Remarkably, for pediatric patients, the parents did not report more, but even significantly lower levels of fatigue. This may be attributable to the usage of the US reference sample (containing both the general population and a clinical sample making it not fully representative for the general population) and to the absence of a representative Dutch reference sample. Higher levels of fatigue have not been previously reported in CG. This reported fatigue may result from a number of causes and may not be CG specific. Fatigue is related to cognitive difficulties including lower information processing speed [32, 33] and also co-exists frequently with anxiety in chronic diseases [34]. Furthermore, again, the Covid-19 pandemic might have magnified the results. Covid-19 related fatigue has been reported in the general population during lockdown measures (“Lockdown fatigue”) [35]. As this study was conducted in the 2–7 months after a (partial) lockdown, the effect of the lockdown fatigue could not be eliminated. Future studies into fatigue levels of CG-patients are warranted.

Lastly, social difficulties were reported by parents of patients with CG. The report of the parents is in line with previous findings of social difficulties [8] and problems with emotion recognition [36], an important component of social cognition. In contrast, pediatric and adult patients themselves did not report any social problems. Remarkably, self-reporting adult patients even reported to have a higher ability to participate in their social roles than the general population. Scores on the adult PROMIS domains of satisfaction with social roles, emotional support, companionship and social isolation did not differ significantly from the general population, indicating no lower satisfaction with social functioning and enough support, companionship and contact with others. In contrast, the generic TAAQOL did indicate social difficulties in adults since the social- and sexuality scale were both significantly decreased. This difference between the PROMIS domains and the TAAQOL might be attributable to the small amount of four questions of the TAAQOL addressing social roles, companionship, social isolation and emotional support, without incorporating the level of social satisfaction. Based on the more reliable PROMIS domains, self-reporting CG-patients are satisfied with their current social activities. This might be due to a larger emphasis on handling social difficulties in follow up of the patients in recent years.

Even though the chosen PROMIS questionnaires largely overlapped between children and adults, pediatric and adult item banks of the same construct do not necessarily measure the construct in an identical manner [37]. Therefore, it is not possible to make exact statements about the difference in self-reported health between pediatric and adult patients with CG. Moreover, due to the presence of only cross-sectional data in the current study and the absence of longitudinal data it is not possible to infer that with age the physical, mental- and social wellbeing of adult patients with CG declines. A recent article reporting on in-depth interviews suggested that the long-term complications of CG are progressive and lead to a decline in HRQoL [38]. However, no longitudinal data was collected in that study and all participating patients were involved in a clinical trial introducing bias towards patients with a more severe outcome. In contrast, longitudinal data consistently showed an absence of a decrease in IQ with age [39]. Although in the current study the older self-reporting adult patients, above the age of 35, reported more difficulties with regard to anxiety, fatigue, physical functioning and cognition than the self-reporting adults between 18 and 35 in comparison to the general population, it must be taken into account that 6 out of 28 patients in this age group, and none in the older group, were unable to complete these questionnaires, and were therefore excluded from the main analyses, skewing the results in the younger group towards a more favorable outcome.

To qualitatively assess these effects of the changes in treatment and follow up since our last HRQoL-study in 2004, possibly benefiting the “younger” group of CG-patients, the generic questionnaires TAPQOL, TACQOL and TAAQOL were re-administered. A limitation of the generic TNO-questionnaires is that it is not possible to link scores on a pediatric questionnaire to scores on an adult questionnaire, in contrast to PROMIS domains using the same metric, allowing the creation of a cross-walk table [40]. Since all pediatric patients participating in the 2004-study are now adults and therefore completed another version of the TNO HRQOL measure, and a new group of pediatric patients was born, no longitudinal analysis was performed. Results of the current pediatric patients on the TAPQOL were comparable to the results in 2004. There were no substantial differences in scale-means. Parents reported stomach problems, and communication difficulties which were now only found in the group of classical phenotype patients. Both parents and children reported lower cognitive functioning on the TACQOL, equal to 2004, but not the lower motor functioning which was previously reported. There were no substantial differences in scale-means. The TAAQOL indicated a lower HRQoL in adults on 7 out of 12 scales. Next to the scales social- and cognitive functioning in 2004, patients now also reported difficulties on the scales gross- and fine motor functioning, sleeping, sexuality and vitality. It is important to take into account that the normative data of the TNO-questionnaires were all collected in the beginning of the 21th century making them less applicable to the current sample in contrast to the sample in the 2004-study. Moreover, the psychometric quality of the PROMIS measures has consistently matched or outperformed generic HRQoL-measures such as the TNO-questionnaires in reliability in both general- and clinical populations while using less items (e.g. [21, 23, 41]).

The secondary aims of the study were to investigate differences in subgroups of patients and to examine the association between HRQoL and intellectual outcome. First, HRQoL of patients diagnosed after newborn- or family screening did not differ significantly from the patients diagnosed after clinical presentation. This is in line with previous findings that there is no difference in intellectual-, language and motor outcomes between classical patients diagnosed by screening and based on clinical presentation [39].

Secondly, there was no significant relation between the pediatric measures and IQ. In adults, patients with a low IQ (< 85) reported significant lower scores on physical-, mental-, and social domains next to lower cognitive functioning. The difference in adult patients with a high and low IQ suggests that the cognitive difficulties may affect other domains, an effect that has been found in other diseases as well [42]. It is possible that this effect is larger in adults, since daily life demands more due to work- and family obligations in adulthood.

### Limitations

The most important limitation of the current study, is the data collection during the Covid-19 pandemic, as all reference data were collected pre-pandemic. The influence of the pandemic has been reduced by performing the data collection 2 months after the end of a partial lockdown. Unfortunately, while we aimed to investigate the entire spectrum of patients with galactosemia it was impossible to administer all questionnaires to the patients with a very low IQ. The effect of selection bias has been reduced by inviting representatives to answer several questionnaires on behalf of the cognitively impaired adult patients. These proxy-reports were treated independently. The exclusion of the severely cognitive affected patients who were unable to complete the questionnaires by themselves from the general analysis, may have caused a positive bias in the same age group of the self-reporting cohort. Furthermore, while Dutch reference samples were used where available, not for all administered PROMIS domains a representative Dutch sample was available. Thus, all parent proxy PROMIS domains except cognition, and the pediatric upper extremity function self-report questionnaire, had reference samples of both the US general population and a clinical sample. The inclusion of a larger group of people with impaired health could lead to an over- or underestimation of the severity of an “average” T-score in the reference sample. Lastly, the sample size was relatively low as is the case in all studies regarding rare diseases. However, the response rate consisted of half of all Dutch patients visiting the expert outpatient clinics making the results generalizable to the entire group. The statistical power issues due to the small sample size were addressed by examining the effect size and not depend solely on the *P* values.

## Conclusions

The current study indicates that even though major changes in treatment and follow-up of CG have been implemented in recent years, CG remains to impact HRQoL of pediatric and adult patients negatively on several domains. The most prevalent effects are on cognition, anxiety, motor function and fatigue. A lower social health was mainly reported by parents, and not by patients themselves. The Covid-19 pandemic might have amplified the results. However, the majority of the results are in line with earlier pre-pandemic findings. The reported higher levels of fatigue need to be monitored and examined in the context of post-pandemic times.

The conclusions of this study ratify the impact of the long-term cognitive-, mental and physical difficulties in CG in all age-groups. It is therefore important for both clinicians and researchers to be attentive to both the pediatric and the adult patients, and the age-dependent difficulties they might encounter.

## Methods

### Patients and recruitment

All patients and/or parents of pediatric patients aged 1 year and older visiting the galactosemia expertise outpatient clinic in either Amsterdam UMC or Maastricht UMC, the Netherlands, were eligible to be invited by regular mail by their treating physician. Patients with an erythrocyte GALT activity < 15% of healthy controls, and/or the presence of two null or severe missense variations in the *GALT* gene were eligible to participate. The limit of quantitation of the GALT enzyme assay in our center was 3.3% (1.1 μmol/h.g Hb). For patients unable to fill out the questionnaires due to cognitive impairments a representative was invited to fill out the questionnaires regarding observable functioning on their behalf. Patients and/or parents unable to fill out the questionnaires due to a language barrier were excluded.

### Procedure

All 26 pediatric patients and parents, and all 48 adult patients visiting the galactosemia expertise outpatient clinic of the Amsterdam UMC (location AMC) were invited. Twenty-one pediatric patients and parents, and 24 of the adult patients visiting the galactosemia expertise outpatient clinic of Maastricht UMC (MUMC) were also invited. Participants signed up for the study by emailing the researchers after which they received personal access codes for the online portal. All questionnaires were available through the research website of the KLIK Patient-Reported Outcome Measures (PROM) portal [43] between the end of June 2021 and the end of November 2021. A digital informed consent form was signed before the questionnaires could be completed. For patients below the age of 8, only one of the parents completed the questionnaires. For patients between the age of 8 and 18, both the patient and one of the parents completed the questionnaires. Parents with multiple children with CG completed separate questionnaires for each child. Adult patients (≥ 18 years) solely completed the questionnaires. For adult patients unable to fill out the questionnaires because of severe cognitive impairment a representative completed the questionnaires regarding observable processes [44]. Medical data including information about the diagnosis, diet and outcome severity (i.e. most recent assessment of IQ and evaluation of movement disorders by a neurologist) were obtained from the treating physician of the patient if available. These data were collected and stored in an electronic clinical report form in Castor Electronic Data Capture (Castor EDC [45]). A waiver was given for the data collection by the Medical Ethics Committee of the Amsterdam UMC.

### Measures

All measures are summarized in Additional file 3: Table S7 for each age group.

#### Socio-demographic questionnaires

Both parents and adult patients filled out a questionnaire with socio-demographic questions about themselves or their child for comparison with the normative group of the PROMIS domains and to investigate educational attainment, social participation and autonomy.

#### PROMIS questionnaires

Item banks and scales, all translated into Dutch, of the patient-reported outcomes measurement information system (PROMIS) were used [41, 46]. Part of the PROMIS domains were administered as Computerized Adaptive Tests (CAT) with standard settings, in which items are selected based on previous responses, resulting in a reliable score with only several items [15]. The other part of the PROMIS domains were administered as short forms, due to the absence of a translated CAT (see Additional file 3). Children and adolescents (8–18 years) completed the following six PROMIS pediatric measures translated into Dutch [47]: CAT V2.0—Anxiety [21, 48], CAT V2.0—Depressive symptoms [21, 48], Short Form V1.0—Cognitive function 7a [49, 50], CAT V2.0—Fatigue [22, 51], CAT V2.0—Upper Extremity [48, 52] and CAT V2.0—Peer relationships [23, 53]. All pediatric measures show good psychometric properties, with anxiety, depressive symptoms, fatigue and peer relationships validated in the Dutch general population, except a moderate stability (α = 0.63) for the upper extremity scale [21–23, 49, 54]. The same measures but adjusted for proxy report were also completed by one of the children’s parents or caregivers if the child was 5 years or older (CAT V2.0—Anxiety [55, 56], CAT V2.0—Depressive symptoms [55, 57], Short Form V1.0—Cognitive function 7a [49, 50], CAT V2.0—Fatigue [55, 58], Short Form V2.0—Upper Extremity 8a [52, 55] and CAT V2.0—Peer relationships [55, 59]). Psychometric properties of the proxy report measures are lacking in the Dutch general population. Ten PROMIS adult measures translated into Dutch [60] were completed by adult patients: CAT V1.0—Anxiety [24, 61, 62], CAT V1.0—Depression [24, 62, 63], Short Form V2.0—Cognitive function [50, 64] 8a, Short Form V1.0—Fatigue 8a [25, 65], CAT V2.0—Physical function [26, 66], CAT V2.0—Ability to participate in social roles and activities [26, 67], CAT V2.0—Satisfaction with social roles and activities [26, 67], Short Form V2.0 Companionship 6a [68, 69], Short Form V2.0—Emotional Support 8a [68, 70] and Short Form V2.0—Social isolation 8a [68, 71]. Anxiety, depression, fatigue, physical function and participation- and satisfaction with social roles were validated in the Dutch general population [24–26]. Representatives of adult patients with severe cognitive disabilities completed the PROMIS adult measures avoiding the items addressing emotions and personal feelings [44]. The recall period of all PROMIS domains is 7 days, except for the pediatric cognitive functioning questionnaire for which the recall period is 4 weeks. A five-point Likert scale is used for scoring the items with differing response categories (e.g. “never”—“(almost) always”, “without any difficulty”—“unable to do”). The total score of the items is converted to a T-score with a mean of 50 and a standard deviation (SD) of 10. A higher score represents more of the measured construct. The resulting T-scores were compared to the best available Dutch or US normative sample.

#### TNO HRQoL questionnaires

The Dutch research institute TNO designed multidimensional questionnaires to assess HRQoL for three different age groups (see Additional file 3). The TNO-AZL Questionnaire for Preschool Children’s Health-Related Quality of Life (TAPQOL [72]) for children between the age of 1 and 6 years consists of 43 items covering 12 scales. The scales communication, motor functioning and social functioning are only applicable to children of 1.5 years and older. All scales were of sufficient psychometric quality (α ≥ 0.50), except for motor functioning with a low reliability coefficient (α = 0.43) [72]. The TNO-AZL Questionnaire for Children’s Health-Related Quality of Life (TACQOL [73, 74]) for children between the age of 6 and 16 years consists of 56 items covering 7 scales. Self-report is only available for children of 8 years and older. All scales were of sufficient psychometric quality. The TNO-AZL Questionnaire for Adult Health-Related Quality of Life (TAAQOL [75]) for (young) adults of 16 years and older consists of 45 items covering 12 scales. All scales were of sufficient psychometric quality. All three questionnaires are generic and multidimensional, measuring several aspects of HRQoL over a time period of respectively the past 3 months, last weeks or the past month. For the TAPQOL and the TAAQOL the scale scores are obtained by adding the item scores within scales and are subsequently linearly transformed to a 0–100 scale. For the TACQOL the scale scores are not transformed. For all questionnaires, a higher score indicates a higher HRQoL. Corresponding normative data, collected more than 20 years ago, available by TNO for the TAPQOL and TAAQOL were used [76]. For the TAAQOL, the age range of the CG-participants was applied to the normative data. For the TACQOL, normative data for the entire age group was no longer available. Therefore, the means and standard deviations of the normative data of the TACQOL of the previous HRQoL-study [10] were used.

### Statistical analysis

All statistical analyses were performed in Rstudio [77]. Patients were split into pediatric (< 18 years) and adult patients. First, descriptive analyses were used to characterize the patients. Second, T-scores of unidimensional PROMIS domains of the self-report of pediatric and adult patients, and the proxy-report of parents were compared to the mean of the corresponding reference group by means of multiple two-sided one-sample t-tests. If the data were not normally distributed, as evaluated by means of the Shapiro–Wilk Test, a nonparametric test (Wilcoxon Signed Rank Test) was used. Correlation analyses were performed to examine the association between age and the T-scores of the unidimensional PROMIS domains and age and the scales of the TNO-questionnaires. If there was a significant association with age, and reference values for different age groups were available, the mean T-scores of the corresponding PROMIS domains were compared to their age appropriate reference mean by means of the above-described analyses. Third, subgroups of patients were compared:

- (1a) classical phenotype pediatric patients (two pathogenic *GALT* mutations and absent or barely detectable erythrocyte GALT activity (< 3.3%) versus (1b) NBS-detected variant pediatric patients (with previously unreported geno- and phenotypes and erythrocyte GALT activity up to 10%, no clinical symptoms at time of diagnosis and undetectable Gal-1-P levels on dietary treatment [2])
- (2a) pediatric and adult patients diagnosed because of clinical symptoms before start of NBS versus (2b) patients diagnosed after family screening and/or NBS.
- (3a) patients with poor intellectual outcome (IQ < 85) versus (3b) good intellectual outcome (IQ ≥ 85). IQ-scores equal or higher than 85 represent scores that are equal or less than one standard deviation of the population mean, representing average intellectual functioning.

Independent t-tests were performed per PROMIS domain to examine differences between groups. If the assumptions (e.g. normal distribution) for the t-tests were not met, a nonparametric test was used (Mann–Whitney Test). Lastly, correlation analyses were performed to examine the association between the T-scores of the unidimensional PROMIS domains and IQ. Outliers were not removed except when a patient consistently reported deviant scores across all domains. Otherwise, it was deemed as part of the range of complications of galactosemia. A *P* value of < 0.05 was considered a statistically significant difference. Since each PROMIS measure is developed to measure one construct, no correction for multiple comparisons was applied. The individual items of the PROMIS short forms were descriptively evaluated to examine possible important items for clinicians evaluating patients with galactosemia. The above described nonparametric tests were used to analyze all TNO-questionnaire scales since the scales were not normally distributed. Cohen’s *d* was used as a statistic for effect size for the parametric tests of the PROMIS domains. An adaption to Cohen’s *d* was used for the non-parametric tests, using the median and median absolute deviation instead of the mean and standard deviation, assuming that the mean and median are equal in the reference samples [78]. An effect size of *d* ≥ 0.20 was considered a small effect, *d* ≥ 0.50 was considered a medium effect and *d* ≥ 0.80 a large effect [79]. For the TNO-questionnaires an equal mean and median in the reference sample could not be assumed. Therefore, Pearson *r* correlation was used as a statistic for effect size. An effect size of *r* ≥ 0.10 was considered a small effect, *r* ≥ 0.30 was considered a medium effect and *r* ≥ 0.50 a large effect [79].

## Supplementary Information

**Additional file 1: Table S1.** Self-reported health of adults with galactosemia on PROMIS domains per age group according to the available reference values for each age group.

**Additional file 2: Tables S2 to S6.** Results of the secondary analyses regarding the differences in HRQoL between patients diagnosed before the introduction of NBS after the presentation of clinical symptoms and patients diagnosed after family screening and/or NBS, and differences in HRQoL between patients with good- and poor intellectual outcome.

**Additional file 3: Table S7.** Administered questionnaires. Complete overview of the administered questionnaires.

## Data Availability

The datasets used and/or analyzed during the current study are available from the corresponding author on reasonable request**.**

## Abbreviations

CAT: Computerized adaptive tests
CG: Classical galactosemia
Gal-1-P: Galactose-1-phosphate
GALT: Galactose-1-phosphate uridyltransferase
HRQoL: Health related quality of life
IQ: Intelligence quotient
NBS: Newborn screening
PROMIS: Patient-reported outcomes measurement information system
TAAQOL: TNO-AZL questionnaire for adult health-related quality of life
TACQOL: TNO-AZL questionnaire for children’s health-related quality of life
TAPQOL: TNO-AZL questionnaire for preschool children’s health-related quality of life
