# Supplementary material for: The challenges of classical galactosemia: HRQoL in pediatric and adult patients
Source: Orphanet J Rare Dis. 2023 Jun 2;18:135. doi: 10.1186/s13023-023-02749-8 (PMC10236383; doi:10.1186/s13023-023-02749-8)
Supplement: Supplementary file 1 — Additional file 1: Table S1. Self-reported health of adults with galactosemia on PROMIS domains per age group according to the available reference values for each age group. [file 13023_2023_2749_MOESM1_ESM.pdf]

Supplementary Table 1. Self-reported health of adults with galactosemia on PROMIS domains per age group according to the available reference values for each age group.

	Galactosemia 18 – 34 years (N = 22)	Reference population 18 – 34 years				Galactosemia 35 – 44 years (N = 5)	Reference population 35 – 44 years				Galactosemia 45 – 54 years (N = 3[@])	Reference population 45 – 54 years			
	Mean (SD)	N	Mean (SD)	*P*	*d*	Mean (SD)	N	Mean (SD)	*P*	*d*	Mean (SD)	N	Mean (SD)	*P*	*d*
Anxiety^	52.0 (6.3)	253	51.8 (9.9)	0.886	0.03	65.4 (11.1)	147	51.4 (10.9)	0.048*	1.26	57.9 (5.9)	173	50.0 (10.9)	0.147	1.34
Depression^	49.1 (7.4)	253	52.0 (9.3)	0.078	0.40	57.5 (6.7)	147	50.5 (10.8)	0.104#	0.79$	57.1 (5.4)	173	50.0 (11.0)	0.149	1.32
Fatigue^	50.8 (10.2)	192	50.3 (9.6)	0.817	0.05	59.6 (10.1)	229	50.6 (10.3)	0.118	0.89	65.7 (4.4)	120	49.4 (11.2)	0.181#	>2.0$
Physical functioning+	53.8 (6.6)	282	55.2 (9.5)	0.350	0.20	48.4 (2.0)	214	52.8 (10.5)	0.008**	2.21	39.3 (10.7)	199	50.0 (11.5)	0.227	1.00
Participation social roles+	55.6 (5.3)	217	51.5 (9.5)	0.002**	0.76	49.6 (6.3)	136	51.6 (8.3)	0.702	0.18	44.9 (11.4)	171	47.5 (10.8)	0.354	0.55
Satisfaction social roles+	48.5 (8.2)	217	48.6 (8.1)	0.976	0.01	48.9 (4.8)	136	45.0 (8.4)	0.145	0.81	42.0 (9.2)	171	46.6 (8.3)	0.474	0.51

N = Sample size. SD = Standard deviation. *P* = P-value. *d* = Cohen's D; ≥ 0.20 = small effect, ≥ 0.50 = medium effect, ≥ 0.80 = large effect. ^ Higher scores indicate more symptoms. +Higher scores indicate better functioning. # Non-parametric test. * P < 0.05, ** P < 0.01, *** P < 0.001. @Except for participation social roles; N=4. $Effect size calculated with median and median absolute deviation.
