# Supplementary material for: The challenges of classical galactosemia: HRQoL in pediatric and adult patients
Source: Orphanet J Rare Dis. 2023 Jun 2;18:135. doi: 10.1186/s13023-023-02749-8 (PMC10236383; doi:10.1186/s13023-023-02749-8)
Supplement: Supplementary file 2 — Additional file 2: Tables S2 to S6. Results of the secondary analyses regarding the differences in HRQoL between patients diagnosed before the introduction of NBS after the presentation of clinical symptoms and patients diagnosed after family screening and/or NBS, and differences in HRQoL between patients with good- and poor intellectual outcome. [file 13023_2023_2749_MOESM2_ESM.pdf]

Supplementary Table 2. Self-, and proxy-reported health in children with galactosemia diagnosed by means of family screening or newborn screening versus children with galactosemia diagnosed after clinical presentation according to both self-report and proxy-report on PROMIS domains.

	Children (age 8 – 18)						Parents or caregivers (age children 5 – 18)					
	FS/NBS		Clinical presentation				FS/NBS		Clinical presentation			
	N	Mean (SD)	N	Mean (SD)	*P*	*d*	N	Mean (SD)		Mean (SD)	*P*	*d*
Anxiety^	12	47.5 (7.5)	3	51.2 (11.5)	0.638	0.45	13	52.3 (7.7)	3	52.0 (10.9)	0.704#	1.07$
Depression^	11	47.5 (9.3)	3	49.0 (12.8)	0.867	0.15	13	46.9 (6.6)	3	46.2 (10.0)	0.924	0.09
Fatigue^	11	46.4 (8.4)	2	41.2 (16.7)	0.735	0.55	13	45.1 (6.6)	2	39.0 (8.1)	0.472	0.90
Cognition+	11	46.3 (5.9)	3	48.5 (5.4)	0.585	0.37	13	45.6 (7.1)	3	49.8 (5.6)	0.328	0.61
Upper extremity function+	11	45.2 (6.8)	2	46.0 (4.9)	0.870	0.11	13	44.9 (10.4)	2	50.4 (6.3)	0.591#	0.23$
Peer relations+	11	48.9 (6.7)	3	43.3 (2.7)	0.056	0.90	13	43.9 (5.0)	3	43.3 (6.2)	0.901	0.10

N = Sample size. SD = Standard deviation. *P* = P-value. *d* = Cohen's D; ≥ 0.20 = small effect, ≥ 0.50 = medium effect, ≥ 0.80 = large effect. ^ Higher scores indicate more symptoms. +Higher scores indicate better functioning. * P < 0.05, ** P < 0.01, *** P < 0.001. $ Effect size calculated with median and median absolute deviation.

Supplementary Table 3. Self-reported health in adults with galactosemia diagnosed by means of family screening versus adults with galactosemia diagnosed after clinical presentation on PROMIS domains.

	Diagnosis after clinical presentation		Diagnosis after family screening			
	N	Mean (SD)	N	Mean (SD)	*P*	*d*
Anxiety^	25	54.3 (8.0)	5	57.3 (11.8)	0.607	0.35
Depression^	25	51.1 (7.6)	5	52.0 (10.1)	0.868	0.10
Fatigue^	25	52.4 (10.5)	5	60.4 (10.9)	0.190	0.75
Cognition+	25	47.1 (9.0)	5	39.6 (13.0)	0.273	0.78
Physical functioning+	25	52.3 (8.2)	5	47.6 (4.7)	0.152#	0.32$
Participation social roles+	26	54.2 (6.8)	5	48.1 (8.8)	0.203	0.86
Satisfaction social roles+	25	48.5 (8.2)	5	45.4 (6.2)	0.372	0.39
Companionship+	25	50.7 (8.1)	5	47.5 (12.4)	0.603	0.36
Emotional support+	25	53.4 (7.9)	5	51.0 (12.8)	0.705	0.27
Social Isolation^	25	46.8 (9.4)	5	55.8 (10.0)	0.120	0.94

N = Sample size. SD = Standard deviation. *P* = P-value. *d* = Cohen's D; ≥ 0.20 = small effect, ≥ 0.50 = medium effect, ≥ 0.80 = large effect. ^ Higher scores indicate more symptoms. +Higher scores indicate better functioning. * P < 0.05, ** P < 0.01, *** P < 0.001. $Effect size calculated with median and median absolute deviation.

Supplementary Table 4. Self-, and proxy-reported health in children with the classical phenotype of galactosemia and good intellectual outcome versus poor intellectual outcome on PROMIS domains.

	Children (age 8 – 18)						Parents or caregivers (age children 5 – 18)					
	Good intellectual outcome		Poor intellectual outcome				Good clinical outcome		Poor clinical outcome			
	N	Mean (SD)	N	Mean (SD)	*P*	*d*	N	Mean (SD)	N	Mean (SD)	*P*	*d*
Anxiety^	5	49.2 (10.1)	4	49.5 (10.8)	0.968	0.03	5	54.9 (8.5)	5	51.4 (13.1)	0.324	0.32
Depression^	5	49.4 (11.0)	4	49.1 (12.4)	0.967	0.03	5	48.6 (4.1)	5	46.6 (10.9)	0.739	0.27
Fatigue^	4	52.3 (6.7)	4	46.2 (11.2)	0.340	0.66	4	46.7 (5.3)	5	43.6 (7.5)	0.524	0.48
Cognition+	5	42.5 (3.3)	4	48.0 (7.7)	0.252	0.99	5	43.1 (5.8)	5	48.2 (8.1)	0.556#	0.24$
Upper extremity function+	4	45.3 (8.7)	4	45.3 (7.8)	0.989	0.01	4	49.8 (10.0)	5	38.8 (11.5)	0.219#	3.02$
Peer relations+	5	48.3 (5.8)	4	42.7 (2.7)	0.104	1.19	5	46.1 (15.4)	5	43.0 (11.5)	0.376	0.65

N = Sample size. SD = Standard deviation. *P* = P-value. *d* = Cohen's D; ≥ 0.20 = small effect, ≥ 0.50 = medium effect, ≥ 0.80 = large effect. ^ Higher scores indicate more symptoms. +Higher scores indicate better functioning. # Non-parametric test. * P < 0.05, ** P < 0.01, *** P < 0.001.
$ Effect size calculated with median and median absolute deviation. Good intellectual outcome: Intelligence Quotient ≥ 85, poor intellectual outcome: Intelligence Quotient < 85.

Supplementary Table 5. Self-reported health in adults with galactosemia and good intellectual outcome versus poor intellectual outcome on PROMIS domains.

	Good intellectual outcome		Poor intellectual outcome			
	N	Mean (SD)	N	Mean (SD)	*P*	*d*
Anxiety^	4	47.8 (5.0)	14	58.3 (8.7)	0.014*	1.30
Depression^	4	43.8 (2.8)	14	54.2 (6.5)	0.003#***	0.67$
Fatigue^	4	43.4 (4.9)	14	58.4 (9.3)	0.001**	1.74
Cognition+	4	57.1 (5.0)	14	41.8 (9.0)	0.001**	1.83
Physical functioning+	4	58.3 (5.4)	14	49.9 (4.9)	0.044*	1.68
Participation social roles+	4	59.3 (3.1)	14	50.9 (7.7)	0.006**	1.19
Satisfaction social roles+	4	55.4 (7.0)	14	49.1 (6.2)	0.336#	0.13$
Companionship+	4	49.3 (4.3)	14	53.2 (8.9)	0.243	0.48
Emotional support+	4	58.5 (6.8)	14	52.9 (9.3)	0.230	0.62
Social Isolation^	4	36.0 (3.9)	14	50.4 (8.9)	0.010#*	1.42$

N = Sample size. SD = Standard deviation. *P* = P-value. *d* = Cohen's D; ≥ 0.20 = small effect, ≥ 0.50 = medium effect, ≥ 0.80 = large effect. ^ Higher scores indicate more symptoms. +Higher scores indicate better functioning. # Non-parametric test. * P < 0.05, ** P < 0.01, *** P < 0.001. $Effect size calculated with median and median absolute deviation. Good intellectual outcome: Intelligence Quotient ≥ 85, poor intellectual outcome: Intelligence Quotient < 85.

Supplementary Table 6. Self-reported HRQoL (TAAQOL) between adults with galactosemia and good intellectual outcome versus poor intellectual outcome.

	Good intellectual outcome		Poor intellectual outcome			
	N	Mean (SD)	N	Mean (SD)	*P*	*r*
Gross motor#	4	87.5 (13.5)	14	76.8 (20.9)	0.362	-0.21
Fine motor#	5	93.8 (8.8)	14	93.3 (11.4)	1.000	0.00
Pain#	4	81.3 (13.5)	14	70.5 (22.1)	0.486	-0.16
Sleeping#	5	53.8 (36.3)	14	50.9 (33.7)	0.963	-0.01
Cognitive function#	5	85.0 (22.4)	14	54.5 (26.3)	0.045*	-0.46
Social function#	5	91.3 (7.1)	14	77.7 (25.3)	0.348	-0.22
Daily activities#	5	72.5 (41.1)	14	70.5 (29.5)	0.708	-0.09
Sexuality#	5	100.0 (0.0)	14	67.9 (28.9)	0.016*	-0.55
Vitality#	5	58.3 (33.3)	14	44.0 (24.1)	0.176	-0.31
Happiness#	5	73.3 (16.0)	14	63.1 (29.4)	0.779	-0.06
Depressive moods#	5	83.3 (16.7)	14	60.7 (29.5)	0.111	-0.37
Agressiveness#	5	97.8 (5.0)	14	88.1 (14.1)	0.059	-0.43

N = Sample size. SD = Standard deviation. *P* = P-value. *r* = Pearson *r* correlation; ≥ 0.10 = small effect, ≥ 0.30 = medium effect, ≥ 0.50 = large effect. Higher scores indicate a better quality of life. * P < 0.05, ** P < 0.01, *** P < 0.001. # Non-parametric test.
