# Supplementary material for: The challenges of classical galactosemia: HRQoL in pediatric and adult patients
Source: Orphanet J Rare Dis. 2023 Jun 2;18:135. doi: 10.1186/s13023-023-02749-8 (PMC10236383; doi:10.1186/s13023-023-02749-8)
Supplement: Supplementary file 3 — Additional file 3: Table S7. Administered questionnaires. Complete overview of the administered questionnaires. [file 13023_2023_2749_MOESM3_ESM.pdf]

Supplementary Table 7. Administered questionnaires

Age	1y – 4y	5y	6y – 7y	8y – 15y	16y – 17y	18y+	18y+ Representative
Socio-demographics	*Parent proxy:*<br>Socio-demographic questionnaire	*Parent proxy:*<br>Socio-demographic questionnaire	*Parent proxy:*<br>Socio-demographic questionnaire	*Parent proxy:*<br>Socio-demographic questionnaire	*Parent proxy:*<br>Socio-demographic questionnaire	*Self:*<br>Socio-demographic questionnaire	*Proxy:*<br>Socio-demographic questionnaire
PROMIS Mental health				*Self:*<br>Anxiety CAT V2.0[*]<br>Depressive symptoms CAT V2.0[*]<br>Cognitive function SF7a V1.0[#]	*Self:*<br>Anxiety CAT V2.0[*]<br>Depressive symptoms CAT V2.0[*]<br>Cognitive function SF7a V1.0[#]	*Self:*<br>Anxiety CAT V1.0[*]<br>Depression CAT V1.0[*]<br>Cognitive function SF8a V2.0[#]	*Proxy:*<br>Cognitive function SF8a V2.0[#]
		Parent proxy:<br>Anxiety CAT V2.0[$]<br>Depressive symptoms CAT V2.0[$]<br>Cognitive function SF7a V1.0[$]	*Parent proxy:*<br>Anxiety CAT V2.0[$]<br>Depressive symptoms CAT V2.0[$]<br>Cognitive function SF7a V1.0[$]	*Parent proxy:*<br>Anxiety CAT V2.0[$]<br>Depressive symptoms CAT V2.0[$]<br>Cognitive function SF7a V1.0[$]	*Parent proxy:*<br>Anxiety CAT V2.0[$]<br>Depressive symptoms CAT V2.0[$]<br>Cognitive function SF7a V1.0[$]		
PROMIS Physical health				*Self:*<br>Physical function CAT V2.0[$]<br>Fatigue CAT V2.0[*]	*Self:*<br>Physical function CAT V2.0[$]<br>Fatigue CAT V2.0[*]	*Self:*<br>Physical function CAT V2.0[*]<br>Fatigue V1.0 SF8a[*]	*Proxy:*<br>Physical function CAT V2.0[*]
		Parent proxy:<br>Physical function UE SF8a V2.0[$]<br>Fatigue CAT V2.0[$]	*Parent proxy:*<br>Physical function UE SF8a V2.0[$]<br>Fatigue CAT V2.0[$]	*Parent proxy:*<br>Physical function UE SF8a V2.0[$]<br>Fatigue CAT V2.0[$]	*Parent proxy:*<br>Physical function UE SF8a V2.0[$]<br>Fatigue CAT V2.0[$]		
PROMIS Social health				*Self:*<br>Peer relationships CAT V2.0[*]	*Self:*<br>Peer relationships CAT V2.0[*]	*Self:*<br>Ability to participate in social roles and activities CAT V2.0[*]<br>Satisfaction with social roles and activities CAT V2.0[*]<br>Companionship SF6a V2.0[#]<br>Emotional support SF8a V2.0[#]<br>Social isolation SF8a V2.0[#]	*Proxy:*<br>Ability to participate in social roles and activities CAT V2.0[*]<br>Companionship SF6a V2.0[#]
		Parent proxy:<br>Peer relationships CAT V2.0[$]	*Parent proxy:*<br>Peer relationships CAT V2.0[$]	*Parent proxy:*<br>Peer relationships CAT V2.0[$]	*Parent proxy:*<br>Peer relationships CAT V2.0[$]		
TNO Questionnaires (and subscales)	*Proxy:*<br>TAPQOL<br>- Sleep<br>- Appetite	*Parent proxy:*<br>TAPQOL<br>- Sleep<br>- Appetite	*Parent proxy:*<br>TACQOL<br>- Physical symptoms	*Self + Parent proxy:*<br>TACQOL<br>- Physical symptoms	*Self:*<br>TAAQOL<br>- Gross motor<br>- Fine motor	*Self:*<br>TAAQOL<br>- Gross motor<br>- Fine motor	

Supplementary Table 7. Administered questionnaires

Age	1y – 4y	5y	6y – 7y	8y – 15y	16y – 17y	18y+	18y+ Representative
	- Lungs<br>- Stomach<br>- Skin<br>- Motor function (≥ 1.5 years)<br>- Social function (≥ 1.5 years)<br>- Problem behavior<br>- Communication (≥ 1.5 years)<br>- Anxiety<br>- Positive mood<br>- Liveliness	- Lungs<br>- Stomach<br>- Skin<br>- Motor function<br>- Social function<br>- Problem behavior<br>- Communication<br>- Anxiety<br>- Positive mood<br>- Liveliness	- Motor function<br>- Autonomy<br>- Cognitive function<br>- Social function<br>- Positive emotions<br>- Negative emotions	- Motor function<br>- Autonomy<br>- Cognitive function<br>- Social function<br>- Positive emotions<br>- Negative emotions	- Pain<br>- Sleeping<br>- Cognitive function<br>- Social function<br>- Daily activities<br>- Sexuality<br>- Vitality<br>- Happiness<br>- Depressive moods<br>- Aggressiveness	- Pain<br>- Sleeping<br>- Cognitive function<br>- Social function<br>- Daily activities<br>- Sexuality<br>- Vitality<br>- Happiness<br>- Depressive moods<br>- Aggressiveness	

Notes. y = years, m = months, PROMIS = Patient-Reported Outcome Measurement Information System, CAT = Computerized Adaptive Tests, SF = Short form, UE = Upper Extremity. [*] Compared to the Dutch general population, [#] Compared to the US general population, [$] Compared to a subset of the US general population and a clinical population.
PROMIS pediatric and proxy-report measures and corresponding normative data: Anxiety – CAT V2.0 (1, 2), Depressive symptoms – CAT V2.0 (1, 3), Cognitive Function – SF7a V1.0 (4), Fatigue – CAT V2.0 (5, 6), Physical Function Upper Extremity (UE) Function – CAT V2.0 and Physical Function Upper Extremity (UE) Function proxy-report SF8a V2.0 (7) and Peer relationships – CAT V2.0 (8, 9).
PROMIS adult measures: Anxiety – CAT V1.0 (10), Depression – CAT V1.0 (10), Cognitive Function – SF8a V2.0 (4), Fatigue – SF8a V1.0 (11), Physical Function – CAT V2.0 (12), Ability to Participate in Social Roles and Activities – CAT V2.0 (12), Satisfaction with Social Roles and Activities – CAT V2.0 (12), Companionship – SF6a V2.0 (13), Emotional Support – SF8a V2.0 (14) and Social isolation – SF8a V2.0 (15)).
TAPQOL = TNO-AZL Questionnaire for Preschool Children's Health-Related Quality of Life (16), TACQOL = TNO-AZL Questionnaire for Children's Health-Related Quality of Life (17), TAAQOL = TNO-AZL Questionnaire for Adult Health-Related Quality of Life (16).

References

1. Klaufus L, Luijten M, Verlinden E, Van der Wal M, Haverman L, Cuijpers P, et al. Psychometric properties of the Dutch-Flemish PROMIS® pediatric item banks Anxiety and Depressive Symptoms in a general population. Qual Life Res. 2021;30(9):2683-95.
2. PROMIS. Anxiety scoring manual. 2022. https://staging.healthmeasures.net/images/PROMIS/manuals/Scoring_Manual_Only/PROMIS_Anxiety_Scoring_Manual_03June2022.pdf. Accessed 01 Sep 2022.
3. PROMIS. Depression scoring manual. 2022. https://staging.healthmeasures.net/images/PROMIS/manuals/Scoring_Manual_Only/PROMIS_Depression_Scoring_Manual_03June2022.pdf. Accessed 01 Sep 2022.
4. PROMIS. Cognitive function scoring manual. 2022. https://staging.healthmeasures.net/images/PROMIS/manuals/Scoring_Manual_Only/PROMIS_Cognitive_Function_Scoring_Manual_03June2022.pdf. Accessed 01 Sep 2022.
5. Peersmann SH, Luijten MA, Haverman L, Terwee CB, Grootenhuis MA, van Litsenburg RR. Psychometric properties and CAT performance of the PROMIS pediatric sleep disturbance, sleep-related impairment, and fatigue item banks in Dutch children and adolescents. Psychol Assess. 2022;34(9):860.
6. PROMIS. Fatigue scoring manual. 2022. https://staging.healthmeasures.net/images/PROMIS/manuals/Scoring_Manual_Only/PROMIS_Fatigue_Scoring_Manual_28June2022.pdf. Accessed 01 Sep 2022.
7. PROMIS. Physical function scoring manual. 2022. https://staging.healthmeasures.net/images/PROMIS/manuals/Scoring_Manual_Only/PROMIS_Physical_Function_Scoring_Manual_26May2022.pdf. Accessed 01 Sep 2022.
8. Luijten MA, van Litsenburg RR, Terwee CB, Grootenhuis MA, Haverman L. Psychometric properties of the Patient-Reported Outcomes Measurement Information System (PROMIS®) pediatric item bank peer relationships in the Dutch general population. Qual Life Res. 2021;30:2061-70.
9. PROMIS. Social relationships (peer, family) scoring manual. 2022. https://staging.healthmeasures.net/images/PROMIS/manuals/Scoring_Manual_Only/PROMIS_Social_Relationships_Scoring_Manual_03June2022.pdf. Accessed 01 Sep 2022.
10. Elsman EB, Flens G, de Beurs E, Roorda LD, Terwee CB. Towards standardization of measuring anxiety and depression: Differential item functioning for language and Dutch reference values of PROMIS item banks. PLoS One. 2022;17(8):e0273287.
11. Terwee CB, Elsman EB, Roorda LD. Towards standardization of fatigue measurement: Psychometric properties and reference values of the PROMIS Fatigue item bank in the Dutch general population. Research Methods in Medicine & Health Sciences. 2022;3(3):86-98.
12. Terwee CB, Roorda LD. Country-specific reference values for PROMIS® pain, physical function and participation measures compared to US reference values. Ann Med. 2023;55(1):1-11.
13. PROMIS. Companionship scoring manual. 2022. https://staging.healthmeasures.net/images/PROMIS/Differences_Between_PROMIS_Measures/PROMIS_Companionship_Measure_Differences_24Jan2022.pdf. Accessed 01 Sep 2022.
14. PROMIS. Emotional support scoring manual. 2021. https://staging.healthmeasures.net/images/PROMIS/manuals/Scoring_Manuals_/PROMIS_Emotional_Support_Scoring_Manual.pdf. Accessed 01 Sep 2022.
15. PROMIS. Social isolation scoring manual. 2021. https://staging.healthmeasures.net/images/PROMIS/manuals/Scoring_Manuals_/PROMIS_Social_Isolation_Scoring_Manual.pdf. Accessed 01 Sep 2022.
16. TNO. Vragenlijsten kwaliteit van leven. 2021. Available from: https://www.tno.nl/media/5004/vragenlijsten_01032012.pdf. Accessed 01 Sep 2022.
17. Bosch AM, Grootenhuis MA, Bakker HD, Heijmans HS, Wijburg FA, Last BF. Living with classical galactosemia: health-related quality of life consequences. Pediatrics. 2004;113(5):e423-8.
